# Clinicopathologic and prognostic implications of HOXA gene and its associated long-noncoding RNAs expression in non-small cell carcinoma: A meta-analysis

**DOI:** 10.1097/MD.0000000000038087

**Published:** 2024-08-09

**Authors:** Yanhui Yang, JinYang Huang, Qi Wang, Ji Li, Lei Yu, Xiaoyang Xie

**Affiliations:** a Department of Thoracic Surgery, The First People’s Hospital of Neijiang, Neijiang Affiliated Hospital of Chongqing Medical University, Neijiang, Sichuan, P.R. China; b Department of Cardiothoracic Surgery, Clinical Medical College of Chengdu Medical College, Chengdu, Sichuan, P.R. China.

**Keywords:** clinicopathological features, HOXA gene, meta-analysis, non-small cell lung cancer, prognosis

## Abstract

**Background:**

We conducted an investigation into the correlation between HOXA and associated long-noncoding RNAs, along with their clinicopathologic and prognostic features in non-small cell lung cancer (NSCLC).

**Methods:**

A comprehensive search across multiple electronic databases, including PubMed and the Web of Science, was conducted to identify relevant studies. The association between HOXA, clinicopathologic parameters, and prognosis was assessed using relative risk (RR) and hazard ratio (HR) with a 95% confidence interval (CI). Data compilation was performed using STATA 12.0 software.

**Results:**

A total of 11 trials involving 2058 patients with NSCLC were included in our study. Significant correlations were observed between HOXA-AS2 and TNM stage (III-IV) (RR=2.173, 95% CI: 1.386–5.437, *P*< 0.05) and HOTTIP and age (≥60-year-old) (RR=2.628, 95% CI: 1.185–5.829, *P*< 0.05) and non-smoking (RR=0.387, 95% CI: 0.156–0.959, P< 0.05). The combined results indicated a significant association between HOXA5 and increased overall survival (HR = 0.69, 95% CI = 0.57–0.84, *P* < .001). Additionally, HOXA-AS2, HOXA11 and HOTTIP were identified as potential independent predictors for poorer OS (HOXA-AS2: HR =3.48, 95% CI = 1.95 to 6.21, P < 0.05; HOXA11: HR=1.39, 95%CI = 1.08 to 1.79, P < 0.05; HOTTIP: HR=2.44, 95%CI = 1.10 to 5.42, P < 0.05). The prognostic significance of HOXA1, HOXA3 and HOXA4 was uncertain (HOXA1: HR=1.40, 95% CI =0.28 to 7.06, P > 0.05; HOXA3: HR=1.20, 95% CI = 0.96 to 1.50, P > 0.05; HOXA4: HR=0.97, 95% CI = 0.77 to 1.23, P > 0.05).

**Conclusions:**

The HOXA gene family has some potential to emerge as a novel prognostic factor for NSCLC and is correlated with some clinicopathological parameters such as the TNM stage, age and smoking. However, further meticulously designed prospective studies are warranted to substantiate these findings.

## 1. Introduction

Non-small cell lung cancer (NSCLC) constitutes 85% of all primary pulmonary carcinomas and stands as the leading cause of cancer-related mortality worldwide.^[1,2]^ Lung cancer, characterized by a complex biological process, arises from the intricate dysregulation of various oncogenes linked to cancer development.^[3]^ Despite advancements in multimodal therapies involving surgery, radiation, and chemotherapy for NSCLC, the 5-year overall survival (OS) rate remains suboptimal.^[4]^ Consequently, the quest for precise and specific indicators to enhance the survival rate in NSCLC remains an imperative undertaking.

Homeobox (HOX) plays a crucial role in embryonic growth and tumor development. Among the 39 HOX genes, HOXA, HOXB, HOXC, and HOXD are located on different chromosomes (7p15.2, 17, 21, 12, 13, 12, 13, and 2, 3.1).^[5]^ The human HOX gene clusters encompass nonprotein-encoding genes, generating numerous long noncoding RNAs (lncRNAs) and evolutionarily conserved microRNAs.^[6,7]^ The nonprotein-encoding RNAs within the HOX clusters play a pivotal role in cancer development by regulating the HOX genes.^[8]^

Studies have indicated a potential correlation between the expression of HOXA genes in NSCLC and tumor invasiveness, patient prognosis, and treatment response. Due to the involvement of HOXA genes in cell differentiation and tumor progression, they emerge as promising targets for future NSCLC therapies. The development of drugs specifically addressing these genes holds the promise of offering more efficacious treatments for NSCLC patients. In-depth exploration of the precise mechanism of action of HOXA genes in NSCLC development can contribute to unveiling the pathogenesis of NSCLC, thereby fostering the formulation of more effective prevention and treatment strategies. Understanding the intricate relationship between HOXA genes and NSCLC establishes a robust foundation for personalized medicine. By scrutinizing the expression of HOXA genes in a patient’s tumor, it may be feasible to tailor treatment plans that align more closely with the patient’s individual conditions.

Thus, we conducted meta-analyses on HOXA and relevant lncRNAs in NSCLC to comprehensively assess their clinical significance, prognostic implications, and therapeutic potential. This rigorous examination aims to unravel the intricate expression patterns of the HOXA gene family and its associated lncRNAs in NSCLC through a meta-analytical approach. Our study is not solely focused on elucidating the fundamental scientific contributions of these molecules to tumor development but, more crucially, on accentuating their significant clinical relevance in advancing the diagnosis, treatment, and prognosis evaluation of NSCLC. Looking forward, the ongoing commitment to expanding our knowledge in this domain is imperative. This dedication plays a pivotal role in advancing therapeutic efficacy and markedly enhancing the quality of life for individuals affected by NSCLC.

## 2. Materials and methods

The present meta-analyses adhere to the Preferred Reporting Items for Systematic Reviews and Meta-Analyses guidelines.^[9]^

### 2.1. Document retrieval strategy

An extensive and systematic search was conducted in 2 electronic databases, namely PubMed and Web of Science, covering the period from November 2014 to May 19, 2023. The search terms “Homeobox A,” “Hoxa,” “Cancer, Non-small Cell Lung,” and “NSCLC” were utilized. Both MeSH terms and free-text phrases were employed to maximize sensitivity.

### 2.2. Criteria for inclusion and exclusion

Classification criteria: (1) NSCLC confirmation through pathology. (2) Subjects classified into HOXA-positive and HOXA-negative based on HOXA expression, and their prognosis compared. (3) Adequate data availability for calculating hazard ratio (HR) with 95% confidence interval (CI) for beneficial outcomes, even if not explicitly mentioned in the essays.

Exclusion criteria: (1) exclusion of letters, reviews, animal studies, case reports, and conference abstracts. (2) In cases of data duplication or overlap, only the most recent publications were considered.

**Figure 1.**
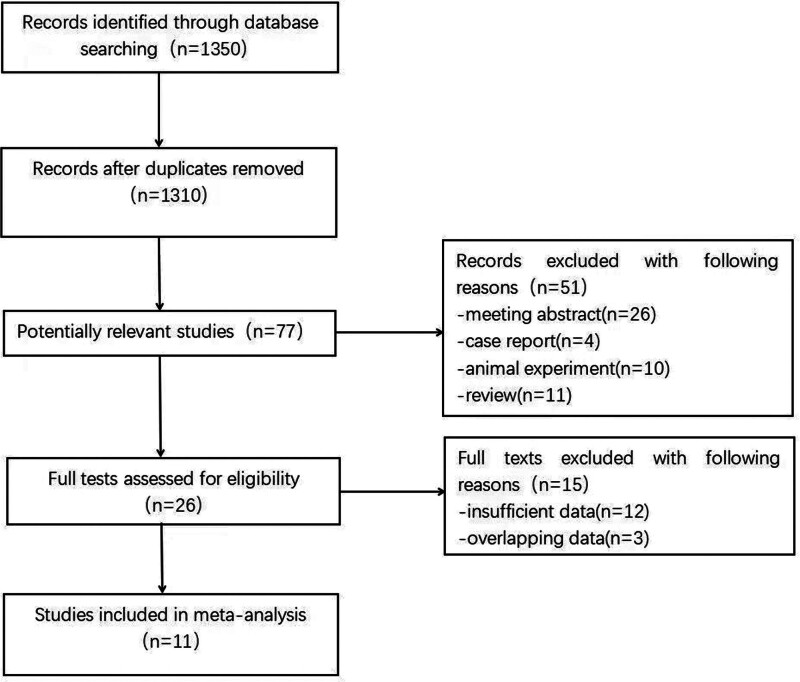
Flow diagram of the literature review.

Document retrieval and selection: 2 independent researchers, Qi Wang and Jinyang Huang, conducted the document retrieval and selection. Any disagreements were resolved through group negotiations until a consensus was reached (Fig. 1).

### 2.3. Extracting data

For each of the mentioned studies, all data have been obtained separately by the 2 authors (Qi Wang and Jinyang Huang): the title of the primary author, the date of release, the country in which the trial was carried out, the size of the sample, HOXA-positive, the tumor-node-metastasis (TNM) phase, and HR with 95% CI for the respective benefit outcomes.

### 2.4. Evaluation of quality

All included studies underwent assessment using the Newcastle-Ottawa Quality Rating Scale (NOS).^[10]^ Studies with a score of 6 or more were considered qualitative. In case–control studies, the NOS scale primarily evaluates 3 aspects: (1) selection of study population, which assesses the representativeness of study subjects and the appropriateness of selecting study and control groups, covering 4 items: definition of the study population, source of study subjects, selection of the control group, and determination of exposure. (2) Assessment of exposure and outcome, focusing on accurately and comprehensively evaluating the exposure and outcome of interest in the study, comprising 2 items: assessment of exposure and assessment of outcome. (3) Comparability of outcomes, which mainly assesses the comparability of outcomes between the study and control groups, including 2 items: analysis of outcomes and interpretation of outcomes.

### 2.5. Analysis of statistics

All statistical analyses were conducted using STATA 12.0. The association between HOXA and clinicopathologic features was assessed through a combined relative risk with a 95% CI, and a combined HR analysis of HOXA and outcomes was performed with a 95% CI. Whenever possible, HR with 95% CI values from multivariate models were utilized. In cases where these values were not directly reported, estimates were derived based on Kaplan–Meier data using the methodology described by Tierney et al.^[11]^ The Chi-square Q test was employed to evaluate the degree of variation among study results. The Q statistic efficiently quantifies heterogeneity by comparing observed and expected variations through chi-square tests. Calculating Q values enabled the identification of statistically significant differences between the results of individual studies. To comprehensively assess the extent and source of heterogeneity, the I² statistical method was further employed. I², a relative measure representing the proportional variation in effect size, serves as a quantitative indicator of heterogeneity degree, ranging from 0% to 100%, with higher I² values indicating greater heterogeneity.^[12]^ A random-effect model was applied in the presence of marked variability (*P* < .10 and/or I² > 50%); otherwise, a fixed-effect model was applied.^[13]^ Subsequently, a subgroup analysis was conducted, stratified by the source of HR, revealing that the source of HR might influence the prognostic value of the HOXA5 gene in NSCLC. The significant association of HOXA5 with OS was observed only in reported sources. Possible publication bias was assessed using Begg funnel plot and Egger test.^[14–16]^ A log-rank *P*-value of < 0.05 was considered significant.

## 3. Results

### 3.1. Document retrieval process

In Figure 1, a total of 1350 records were retrieved from both databases. After removing 40 duplicates, the titles and abstracts of 1310 records were scrutinized. Among these, 51 entries from 77 relevant publications were excluded based on the following criteria: occurrence summary (n = 26), event (n = 4), animal trial (n = 10), and revision (n = 11). Subsequently, 26 full texts were assessed for eligibility, with 15 being excluded due to insufficient data (n = 12) and data duplication (n = 3). Ultimately, 11 trials involving 2058 subjects met the eligibility criteria and were included in the systematic review and meta-analysis (Fig. 1).^[17–27]^

### 3.2. Key features of the covered studies

The involved trials were all retrospective with a NOS rating of 6 or above. Among the 11 trials included, the majority (10/11) were conducted in China. The percentage of subjects exhibiting HOXA expression ranged from 29.4% to 65.3%, and the sample sizes varied between 10 and 719. Furthermore, the HOXA expression state was assessed through immunohistochemistry in all the trials included. More detailed features are presented in Table 1.

**Table 1:** Basic characteristics of included studies.

Author	Year	Country	Sample size	Positive, n (%)	TNM stage	Detection method	Outcome	Source of HR		NOS score	Gene
Yanhua Li^[17]^	2017	China	103	52 (50.5)	I–Ⅳ	IHC	OS + DFS	R	6		HOXA-AS2
Xia Yang^[18]^	2018	China	343	224 (65.3)	I–Ⅳ	IHC	OS	R	7		HOXA11
Chi-Jen Chang^[19]^	2017	China	71	26 (36.6)	I–Ⅳ	IHC	OS	E	7		HOXA5
Alfons Navarro^[20]^	2019	Spanish	99	43 (43.4)	I–Ⅱ	IHC	OS	E	7		HOTTIP
Yue Li^[21]^	2020	China	79	29 (36.7)	I–Ⅳ	IHC	OS	R	6		HOXA5
Fen Zhao^[22]^	2022	China	10	5 (50)	NR	IHC	OS	R	8		HOXA1
Chi-Chung Wang^[23]^	2015	China	68	20 (29.4)	I–Ⅲ	IHC	OS	R	7		HOXA5
T-J Cui^[24]^	2019	China	40	20 (50)	I–Ⅳ	IHC	OS	R	7		HOXA-AS2
Bin-Liang Gan^[25]^	2018	China	475	236 (49.7)	I–Ⅳ	IHC	OS + DFS	R	8		HOXA3
Mei-Ling Zhang^[26]^	2014	China	51	28 (54.9)	IA–ⅢA	IHC	OS + DFS	R	6		HOXA5
Li Gao^[27]^	2022	China	719	354 (49.2)	NR	IHC	OS	E	7		HOXA4
Li Gao^[27]^	2022	China	719	357 (49.7)	NR	IHC	OS	E	7		HOXA5

CRT = chemoradiotherapy, DFS = disease-free survival, E = estimated, HR = hazard ratio, Mixed = surgery and (or) chemoradiotherapy, NOS = Newcastle-Ottawa quality assessment scale, NR = not reported, OS = overall survival, R = reported, Surg = surgery, TNM = tumor-node-metastasis.

### 3.3. Relationship between HOXA and clinicopathologic parameters in NSCLC

The study explored the correlation between HOXA and various parameters, encompassing gender, age (*≥*65 vs <65 years), lymphatic metastasis, TNM stage (IV, III vs II, I), and smoking history. Significant correlations were observed between HOXA-AS2 and TNM stage (III-IV) (RR=2.173, 95% CI: 1.386–5.437, P< 0.05) and HOTTIP and age (≥60-year-old) (RR=2.628, 95% CI: 1.185–5.829, *P*< 0.05) and non-smoking (RR=0.387, 95% CI: 0.156–0.959, *P*< 0.05), as detailed in Table 2.

**Table 2:** Associations of HOXA expression with clinicopathological characteristics in non-small cell lung cancer.

Author	Sex (M vs F)	Age (≥60 vs <60)	Lymph node metastasis (N + vs -)	TNM (IV, III vs II, I)	Smoking history (yes vs no)
Yanhua Li	1.391 (0.625–2.816)	1.211 (0.491–2.098)	2.061 (0.774–5.038)	2.173 (1.386–5.437), P＜0.05	1.783 (0.920–3.457)
Xia Yang	-	-	-	-	-
Bin-Liang Gan	-	-	-	-	-
Alfons Navarro	3.748 (0.889–15.8)	2.628 (1.185–5.829), P＜0.05	-	-	0.387 (0.156–0.959), P＜0.05
T-J Cui	-	-	-	-	-
Fen Zhao	-	-	-	-	-
Li Gao	-	-	-	-	-
Chi-Jen Chang	-	-	-	-	-
Chi-Chung Wang	-	-	-	-	-
Mei-Ling Zhang	-	-	-	-	-
Yue Li	-	-	-	-	-
					

F = female, HOXA5 = homeobox A5, M = male, TNM = tumor-node-metastasis.

### 3.4. Association of HOXA with prognosis in NSCLC

**Figure 2.**
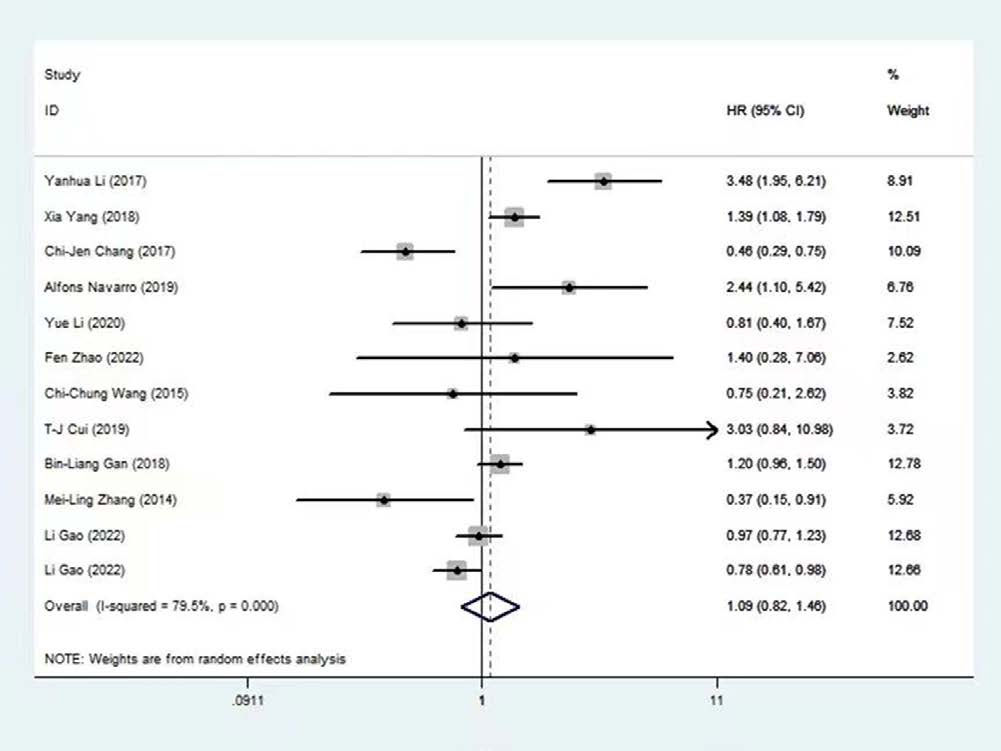
Forest plot of the association between HOXA gene and its associated long-noncoding RNAs and overall survival.

**Figure 3.**
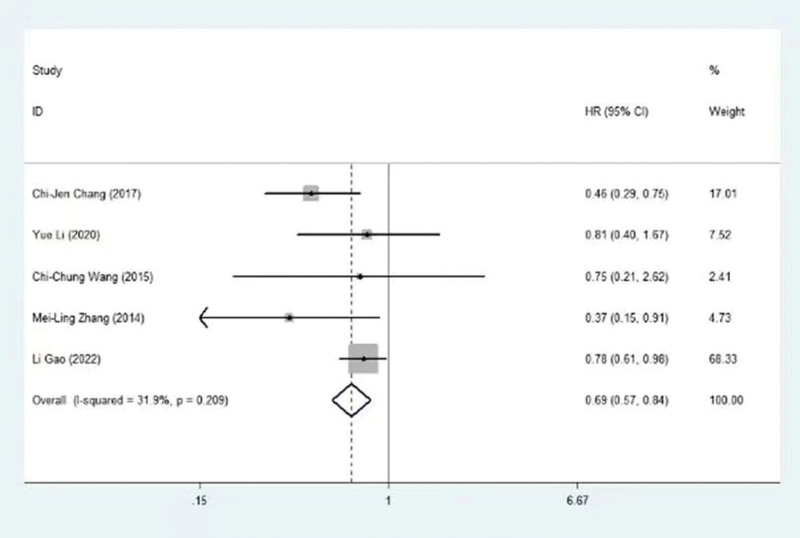
Forest plot of the association between HOXA5 gene and overall survival.

The association between HOXA5 and OS was identified in 5 trials, encompassing 988 patients. In Figure 2, we can see the relationship between the HOXA gene and its associated lncRNAs and overall survival. Moreover, the combined data revealed that HOXA5 expression was correlated with prolonged survival in NSCLC (HR = 0.69, 95% CI = 0.57–0.84, *P* < .001), with nonsignificant heterogeneity (I2 = 31.9%, *P* = .209) (Table 3 and Fig. 3). Additionally, HOXA-AS2, HOXA11 and HOTTIP were suggested as independent predictive factors associated with poorer OS (HOXA-AS2:HR =3.48, 95% CI = 1.95 to 6.21, *P* < 0.05; HOXA11: HR=1.39,95% CI = 1.08 to 1.79, *P* < 0.05; HOTTIP:HR=2.44, 95% CI = 1.10 to 5.42, *P* < 0.05). The prognostic significance of HOXA1, HOXA3 and HOXA4 is unclear (HOXA1: HR=1.40, 95% CI =0.28 to 7.06, *P* > 0.05; HOXA3: HR=1.20, 95% CI = 0.96 to 1.50, *P* > 0.05; HOXA4: HR=0.97, 95% CI = 0.77 to 1.23, *P* >0.05). These findings were consistently supported by subgroup analyses stratified by the origin of HR. Refer to the detailed results in Table 3 and Table 4

**Table 3:** Meta-analyses for the association of HOXA5 expression with survival of non-small cell lung cancer.

Analysis	No. of studies	HR (95% CI)	Log-rank *P*-value	I² (%)	*P*-value
Overall survival	5	0.64 (0.47,0.87)	.209	31.9	.004
Source of HR Estimated	2	0.63 (0.38,1.04)	.051	73.1	.071
Reported	3	0.62 (0.37,1.04)	.390	0.0	.068

CI = confidence interval, HOXA5 = homeobox A5, HR = hazard ratio.

**Table 4:** The association of HOXA-AS2, HOXA11,HOTTIP, HOXA1, HOXA3 and HOXA4 expression with survival of non-small-cell lung cancer.

Author	Year	Gene	Hazard ratio	Low limit	Upper limit
Yanhua Li	2017	HOXA-AS2	3.48	1.95	6.21
Xia Yang	2018	HOXA11	1.39	1.08	1.79
Alfons Navarro	2019	HOTTIP	2.442	1.100	5.418
Fen Zhao	2022	HOXA1	1.4	0.28	7.06
T-J Cui	2019	HOXA-AS2	3.03	0.84	10.98
Bin-Liang Gan	2018	HOXA3	1.20	0.96	1.50
Li Gao	2022	HOXA4	0.97	0.77	1.23

**Figure 4.**
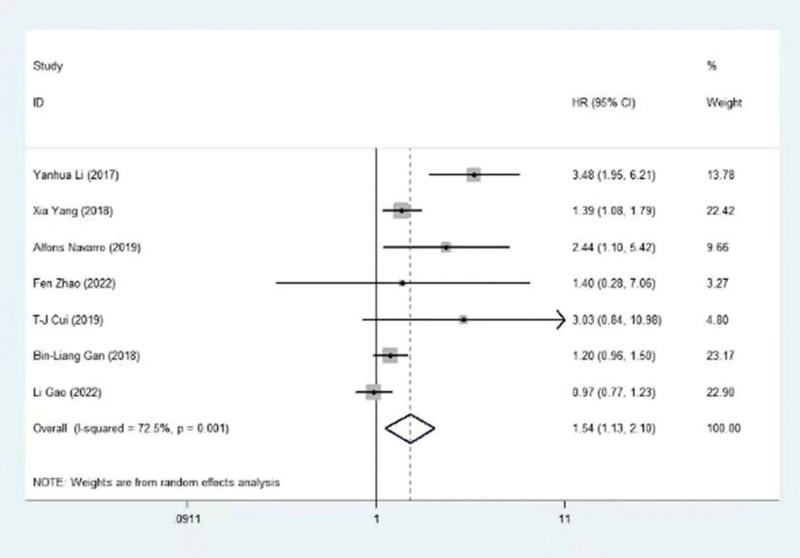
Begg’s funnel plot of the association between HOXA gene and its associated long non-coding RNAs and overall survival.

### 3.5. Publication bias

We conducted Begg’s Funnel Plot and Egger’s Test to assess potential publication bias. In the absence of bias, these points should be symmetrically distributed on both sides of the funnel. Asymmetries or missing points may indicate publication bias. A significant slope in the regression line suggests an abnormal association between the effect size and standard error, indicating publication bias. The Begg’s funnel plot exhibits symmetry, and Egger’s test yields a P-value of 0.399 (overall efficacy) (Figure 4).

## 4. Discussion

To date, research has demonstrated the crucial involvement of HOXA and associated lncRNAs in the development and advancement of various cancer types, including breast cancer and NSCLC. Our study posits that the HOXA5 gene may function as a tumor suppressor in NSCLC.

HOX transcription factors play a crucial role in regulating signaling pathways that control organ morphogenesis and maintain cell fate and differentiation in adults.^[14,16]^ Recent research has delved into the exploration of Hox genes’ involvement in organogenesis, with a specific focus on the lung.^[28, 29]^ The underlying mechanism of HOXA5 in lung growth appears to be modulated by Notch signaling.^[30]^ HOXA5, implicated not only in normal pulmonary growth but also in tumor formation, has been the subject of numerous studies indicating a potential association between HOXA5 methylation and prognostic factors.^[31–33]^ Notably, methylation of the HOXA5 promoter is detected in pulmonary carcinoma,^[34]^ with approximately 81.3% of NSCLC cases exhibiting methylated HOXA5 promoter. Additionally, RT-PCR and immunohistochemistry have revealed a correlation between HOXA5 methylation and gene expression.^[35]^ Our findings thus far indicate higher expression levels of HOXA5 in less-aggressive cells compared to their more aggressive counterparts. Furthermore, the overexpression of HOXA5 has been shown to suppress the invasion capacity of pulmonary carcinoma cells.^[36]^ Consequently, we posit that HOXA5 may serve as a potent inhibitor of NSCLC.

Recently, evidence has emerged suggesting a collaborative role of HOXA5 and p53 in suppressing the invasion of NSCLC. Immunohistochemistry assays for p53 and HOXA5 conducted on primary NSCLC samples revealed that patients exhibiting no immune response to either p53 or HOXA5 experienced unfavorable outcomes.^[37]^ Notably, the overexpression of HOXA5 in lung adenocarcinoma cells demonstrated a restraining effect on proliferation, invasion, and filopodia proliferation, along with the inhibition of in vivo metastasis. Conversely, the knockout of HOXA5 was associated with increased invasion of pulmonary carcinoma cells.^[36]^ Epigenetic regulation has been identified as a key factor governing HOX expression, with methylation playing a pivotal role in HOX silencing. Numerous studies have reported the occurrence of CpG methylation in HOX genes during breast carcinogenesis.^[38–40]^

The Hox transcript antisense intergenic RNA (HOTTIP) is situated on the 5’ side of the HOXA group and is transcribed as a functional lncRNA. Numerous studies have reported the dysregulation of the lncRNA HOTTIP in various tumors, including pulmonary carcinoma.^[41–51]^ In small cell lung cancer (SCLC), HOTTIP expression has been correlated with clinical staging, survival rates, and responses to chemotherapy.^[52, 53]^ Through the modulation of the miR-574-5p/EZH1 axis, overexpression of HOTTIP has been shown to enhance SCLC cell proliferation and cell cycle progression.^[53]^ Moreover, HOTTIP regulates the apoptotic activity and chemoresistance of SCLC cells by interacting with miR-216a, thereby increasing BCL-2 expression and inhibiting apoptosis.^[52]^ In addition to its role in SCLC, HOTTIP exhibits elevated expression in NSCLC, where its overexpression promotes cell proliferation and migration. Notably, HOTTIP overexpression also negatively regulates HOXA13.^[54]^ Furthermore, the overexpression of HOTTIP contributes to drug resistance in lung adenocarcinoma through the modulation of AKT signaling pathways.^[55]^ Collectively, these findings suggest that HOTTIP, as a lncRNA, serves as a crucial marker in the development of pulmonary carcinoma.

In the context of NSCLC, HOXA genes play crucial roles in multiple biological processes relevant to tumor development and progression. Firstly, HOXA genes exert regulatory control over cell proliferation and apoptosis, pivotal processes in tumor growth and maintenance. Certain HOXA genes have demonstrated associations with apoptosis inhibition and the promotion of cell proliferation in NSCLC cells. Secondly, HOXA genes influence the tumor microenvironment by modulating the expression of genes involved in angiogenesis, invasion, and metastasis. This modulation significantly impacts the ability of NSCLC cells to disseminate and form secondary tumors. Thirdly, HOXA genes serve as targets for epigenetic regulation, including DNA methylation and histone modification, leading to altered expression in cancer cells. Epigenetic silencing of specific HOXA genes has been reported in NSCLC, contributing to tumor progression. Fourth, HOXA genes participate in various signaling pathways crucial for NSCLC development, such as the Wnt signaling pathway. The interplay between HOXA genes and these pathways influences tumor cell behavior and responses to therapeutic interventions. Fifth, HOXA genes are implicated in the regulation of cancer stem cells, believed to be responsible for tumor recurrence and treatment resistance. Certain HOXA genes’ expression is linked to the maintenance of stem cell-like properties in NSCLC cells.

In terms of clinical significance, the aberrant expression of HOXA genes may function as a molecular marker for NSCLC, facilitating the identification of tumors with invasive growth potential. Elevated expression of HOXA genes correlates with poor prognosis in NSCLC patients, making it a potential biomarker for prognosis assessment. Given the pivotal role of HOXA genes in tumor development, they emerge as potential therapeutic targets for NSCLC, particularly in patients lacking conventional treatment targets. Future research avenues can be delineated as follows: delve deeper into the specific mechanisms of HOXA genes in NSCLC, exploring their impact on the tumor microenvironment and interactions with other molecular pathways; employ gene-editing techniques like CRISPR/Cas9 to precisely edit HOXA genes and unravel their function in tumor development; assess the safety and efficacy of targeted therapies against HOXA genes in animal models; conduct clinical trials for NSCLC patients with HOXA gene mutations to evaluate the clinical benefits of targeted therapies; explore the synergistic application of HOXA gene inhibitors with other treatment modalities, such as immunotherapy, to enhance therapeutic effectiveness. Real-world applications include offering personalized treatment recommendations based on the expression status of HOXA genes, selecting the most appropriate treatment strategy; developing inhibitors targeting HOXA genes to introduce novel treatment options for NSCLC patients; monitoring disease progression and therapeutic efficacy through regular detection of HOXA gene expression levels; integrating HOXA gene testing into the molecular diagnostic panel for NSCLC, aligning with the goals of precision medicine.

It is concluded that HOXA genes, along with other lncRNAs, show promise in predicting the outcome of pulmonary carcinoma. Moreover, additional research is warranted to ascertain whether HOXA and related lncRNAs might exert a substantial impact on the prognosis in different subtypes of NSCLC.

## 5. Limitations

The meta-analysis is subject to several limitations. Firstly, all included studies are retrospective with relatively small sample sizes, introducing a potential bias. Secondly, due to the absence of stratified data in the original studies, we were unable to conduct subgroup analyses based on age, sex, TNM, or other prognostic factors. Thirdly, while our results did not indicate apparent bias, there may be inherent bias as we only considered English-language publications. Fourthly, a majority of the studies originated from Asia, introducing a potential regional bias. Therefore, it is imperative to conduct additional research in non-Asian populations to validate the findings of the meta-analyses.

## 6. Conclusion

Hence, the HOXA gene family has some potential to serve as a novel prognostic factor for NSCLC and is correlated with some clinicopathological parameters including the TNM stage, age and smoking. However, substantiating our results would necessitate a larger number of prospective trials.

## Acknowledgments

Yanhui Yang and Xiaoyang Xie made the significant contributions to this project’s conception and design, while Qi Wang and Jinyang Huang looked for information, picked out information, Qi Wang and Jinyang Huang wrote the draft, and Ji Li and Yulei edited the thesis and did some statistics. The final draft has been read and approved by all the authors.

## Author contributions

**Conceptualization:** Xiaoyang Xie.

**Data curation:** Yanhui Yang, JinYang Huang, Qi Wang, Lei Yu.

**Formal analysis:** Yanhui Yang, JinYang Huang, Qi Wang, Ji Li, Lei Yu.

**Funding acquisition:** Xiaoyang Xie.

**Investigation:** JinYang Huang, Ji Li, Lei Yu.

**Methodology:** JinYang Huang, Qi Wang, Ji Li.

**Project administration:** Ji Li.

**Resources:** JinYang Huang, Qi Wang, Ji Li, Lei Yu.

**Software:** JinYang Huang, Qi Wang, Ji Li, Lei Yu.

**Supervision:** Yanhui Yang, Ji Li.

**Writing – original draft:** JinYang Huang, Qi Wang.

**Writing – review & editing:** Yanhui Yang, JinYang Huang, Qi Wang, Lei Yu.

## References

[1] Ettinger DS Akerley W Borghaei H. Non-small cell lung cancer. J Natl Compr Canc Netw. 2012;10:1236–71. 23054877 10.6004/jnccn.2012.0130

[2] Jemal A Bray F Center MM Ferlay J Ward E Forman D. Global cancer statistics. CA Cancer J Clin. 2011;61:69–90. 21296855 10.3322/caac.20107

[3] Goldstraw P Ball D Jett JR. Non-small-cell lung cancer. Lancet. 2011;378:1727–40. 21565398 10.1016/S0140-6736(10)62101-0

[4] Molina JR Yang P Cassivi SD Schild SE Adjei AA. Non-small cell lung cancer: epidemiology, risk factors, treatment, and survivorship. Mayo Clin Proc. 2008;83:584–94. 18452692 10.4065/83.5.584 PMC2718421

[5] Apiou F Flagiello D Cillo C Malfoy B Poupon MF Dutrillaux B. Fine mapping of human HOX gene clusters. Cytogenet Cell Genet. 1996;73:114–5. 8646877 10.1159/000134320

[6] Fantini S Salsi V Zappavigna V. HOX cluster-embedded micro-RNAs and cancer. Biochim Biophys Acta Rev Cancer. 2018;1869:230–47. 29540308 10.1016/j.bbcan.2018.03.002

[7] Rinn JL Kertesz M Wang JK. Functional demarcation of active and silent chromatin domains in human HOX loci by noncoding RNAs. Cell. 2007;129:1311–23. 17604720 10.1016/j.cell.2007.05.022 PMC2084369

[8] Botti G De Chiara A Di Bonito M. Noncoding RNAs within the HOX gene network in tumor pathogenesis and progression. J Cell Physiol. 2018;234:395–413. 30132877 10.1002/jcp.27036

[9] Moher D Shamseer L Clarke M. Preferred reporting items for systematic review and meta-analysis protocols (PRISMA-P) 2015 statement. Syst Rev. 2015;4:1. 25554246 10.1186/2046-4053-4-1 PMC4320440

[10] Stang A. Critical evaluation of the Newcastle-Ottawa scale for the assessment of the quality of nonrandomized studies in meta-analyses. Eur J Epidemiol. 2010;25:603–5. 20652370 10.1007/s10654-010-9491-z

[11] Tierney JF Stewart LA Ghersi D Burdett S Sydes MR. Practical methods for incorporating summary time-to-event data into meta-analysis. Trials. 2007;8:16. 17555582 10.1186/1745-6215-8-16 PMC1920534

[12] Lau J Ioannidis JP Schmid CH. Quantitative synthesis in systematic reviews. Ann Intern Med. 1997;127:820–6. 9382404 10.7326/0003-4819-127-9-199711010-00008

[13] Zintzaras E Ioannidis JP. HEGESMA: genome search meta-analysis and heterogeneity testing. Bioinformatics. 2005;21:3672–3. 15955784 10.1093/bioinformatics/bti536

[14] Mandeville I Aubin J LeBlanc M. Impact of the loss of Hoxa5 function on lung alveogenesis. Am J Pathol. 2006;169:1312–27. 17003488 10.2353/ajpath.2006.051333 PMC1698857

[15] Peters JL Sutton AJ Jones DR Abrams KR Rushton L. Comparison of two methods to detect publication bias in meta-analysis. JAMA. 2006;295:676–80. 16467236 10.1001/jama.295.6.676

[16] Volpe MV Wang KT Nielsen HC Chinoy MR. Unique spatial and cellular expression patterns of Hoxa5, Hoxb4, and Hoxb6 proteins in normal developing murine lung are modified in pulmonary hypoplasia. Birth Defects Res A Clin Mol Teratol. 2008;82:571–84. 18553509 10.1002/bdra.20481 PMC2670891

[17] Li Y Jiang H. Up-regulation of long non-coding RNA HOXA-AS2 in non-small cell lung cancer is associated with worse survival outcome. Int J Clin Exp Pathol. 2017;10:9690–6. 31966850 PMC6965976

[18] Yang X Deng Y He R. Upregulation of HOXA11 during the progression of lung adenocarcinoma detected via multiple approaches. International Journal of Molecular Medicine. 2018. 10.3892/ijmm.2018.3826 PMC6192730 30106131

[19] Chang C Chen Y Hsieh C. HOXA5 and p53 cooperate to suppress lung cancer cell invasion and serve as good prognostic factors in non-small cell lung cancer. Journal of Cancer 2017;8:1071–81 28529621 10.7150/jca.17295 PMC5436261

[20] Navarro A Moises J Santasusagna S. Clinical significance of long non-coding RNA HOTTIP in early-stage non-small-cell lung cancer. BMC Pulmonary Medicine 2019;19 10.1186/s12890-019-0816-8 PMC6393998 30819158

[21] Li Y Cui L Li H. The relationship of EZH2 and HOXA5 with non-small cell lung carcinoma patient survival rate. Translational Cancer Research 2020;9:1761–7 35117523 10.21037/tcr.2020.03.37 PMC8798847

[22] Zhao F Tian H Liu X. Homeobox A1 Facilitates Immune Escape and Alleviates Oxidative Stress in Lung Adenocarcinoma. Oxidative Medicine and Cellular Longevity 2022;2022:1–34 10.1155/2022/4102666 PMC9136634 35633885

[23] Wang C Singh S Su K. HOXA5 inhibits metastasis via regulating cytoskeletal remodelling and associates with prolonged survival in non-small-cell lung carcinoma. PLos One. 2015;10:e0124191. 25875824 10.1371/journal.pone.0124191 PMC4396855

[24] Cui TJ Lin GS Dai YM. LncRNA HOXA-AS2 regulates microRNA-216a-5p to promote malignant progression of non-small cell lung cancer. Eur Rev Med Pharmacol Sci. 2019;23(3 Suppl):264–73. 31389597 10.26355/eurrev_201908_18656

[25] Gan BL He RQ Zhang Y. Downregulation of HOXA3 in lung adenocarcinoma and its relevant molecular mechanism analysed by RT-qPCR, TCGA and in silico analysis. Int J Oncol. 2018;53:1557–79. 30066858 10.3892/ijo.2018.4508 PMC6086630

[26] Zhang ML Nie FQ Sun M. HOXA5 indicates poor prognosis and suppresses cell proliferation by regulating p21 expression in non small cell lung cancer. Tumour Biol. 2015;36:3521–31. 25549794 10.1007/s13277-014-2988-4

[27] Gao L He R Huang Z. Expression Landscape and Functional Roles of HOXA4 and HOXA5 in Lung Adenocarcinoma. International Journal of Medical Sciences 2022;19:572–87 35370463 10.7150/ijms.70445 PMC8964330

[28] Boucherat O Montaron S Bérubé-Simard FA. Partial functional redundancy between Hoxa5 and Hoxb5 paralog genes during lung morphogenesis. Am J Physiol Lung Cell Mol Physiol. 2013;304:L817–30. 23585229 10.1152/ajplung.00006.2013 PMC3680751

[29] Kinkead R LeBlanc M Gulemetova R. Respiratory adaptations to lung morphological defects in adult mice lacking Hoxa5 gene function. Pediatr Res. 2004;56:553–62. 15295088 10.1203/01.PDR.0000139427.26083.3D

[30] Boucherat O Chakir J Jeannotte L. The loss of Hoxa5 function promotes Notch-dependent goblet cell metaplasia in lung airways. Biol Open. 2012;1:677–91. 23213461 10.1242/bio.20121701 PMC3507293

[31] Loh M Liem N Vaithilingam A. DNA methylation subgroups and the CpG island methylator phenotype in gastric cancer: a comprehensive profiling approach. BMC Gastroenterol. 2014;14:55. 24674026 10.1186/1471-230X-14-55 PMC3986689

[32] Watson RE Curtin GM Hellmann GM Doolittle DJ Goodman JI. Increased DNA methylation in the HoxA5 promoter region correlates with decreased expression of the gene during tumor promotion. Mol Carcinog. 2004;41:54–66. 15352125 10.1002/mc.20043

[33] Yoo KH Park YK Kim HS Jung WW Chang SG. Epigenetic inactivation of HOXA5 and MSH2 gene in clear cell renal cell carcinoma. Pathol Int. 2010;60:661–6. 20846263 10.1111/j.1440-1827.2010.02578.x

[34] Abe M Hamada J Takahashi O. Disordered expression of HOX genes in human non-small cell lung cancer. Oncol Rep. 2006;15:797–802. 16525661

[35] Kim DS Kim MJ Lee JY. Epigenetic inactivation of Homeobox A5 gene in nonsmall cell lung cancer and its relationship with clinicopathological features. Mol Carcinog. 2009;48:1109–15. 19554572 10.1002/mc.20561

[36] Wang CC Su KY Chen HY. HOXA5 inhibits metastasis via regulating cytoskeletal remodelling and associates with prolonged survival in non-small-cell lung carcinoma. PLoS One. 2015;10:e0124191. 25875824 10.1371/journal.pone.0124191 PMC4396855

[37] Chang CJ Chen YL Hsieh CH. HOXA5 and p53 cooperate to suppress lung cancer cell invasion and serve as good prognostic factors in non-small cell lung cancer. J Cancer. 2017;8:1071–81. 28529621 10.7150/jca.17295 PMC5436261

[38] Raman V Martensen SA Reisman D. Compromised HOXA5 function can limit p53 expression in human breast tumours. Nature. 2000;405:974–8. 10879542 10.1038/35016125

[39] Rawat VP Thoene S Naidu VM. Overexpression of CDX2 perturbs HOX gene expression in murine progenitors depending on its N-terminal domain and is closely correlated with deregulated HOX gene expression in human acute myeloid leukemia. Blood. 2008;111:309–19. 17855634 10.1182/blood-2007-04-085407

[40] Tommasi S Karm DL Wu X Yen Y Pfeifer GP. Methylation of homeobox genes is a frequent and early epigenetic event in breast cancer. Breast Cancer Res. 2009;11:R14. 19250546 10.1186/bcr2233 PMC2687719

[41] Chen X Han H Li Y Zhang Q Mo K Chen S. Upregulation of long noncoding RNA HOTTIP promotes metastasis of esophageal squamous cell carcinoma via induction of EMT. Oncotarget. 2016;7:84480–5. 27806322 10.18632/oncotarget.12995 PMC5356674

[42] Gao W Wu XL Li DZ Liu HD. HOTTIP participates in mammary cancer by promoting cell proliferation via PI3K/AKT pathway. Eur Rev Med Pharmacol Sci. 2018;22:4181–7. 30024606 10.26355/eurrev_201807_15411

[43] Guan Q Zhang Q Zhang C Liu Q Ren QL. HOTTIP regulates progression of endometrial cancer via activating PI3K/AKT pathway. Eur Rev Med Pharmacol Sci. 2018;22:3727–33. 29949146 10.26355/eurrev_201806_15252

[44] Lian Y Cai Z Gong H Xue S Wu D Wang K. HOTTIP: a critical oncogenic long non-coding RNA in human cancers. Mol Biosyst. 2016;12:3247–53. 27546609 10.1039/c6mb00475j

[45] Lin C Wang Y Wang Y. Transcriptional and posttranscriptional regulation of HOXA13 by lncRNA HOTTIP facilitates tumorigenesis and metastasis in esophageal squamous carcinoma cells. Oncogene. 2017;36:5392–406. 28534516 10.1038/onc.2017.133

[46] Peng F Shi X Meng Y. Long non-coding RNA HOTTIP is upregulated in renal cell carcinoma and regulates cell growth and apoptosis by epigenetically silencing of LATS2. Biomed Pharmacother. 2018;105:1133–40. 30021349 10.1016/j.biopha.2018.06.081

[47] Sun Y Zeng C Gan S. LncRNA HOTTIP-Mediated HOXA11 expression promotes cell growth, migration and inhibits cell apoptosis in breast cancer. Int J Mol Sci . 2018;19:472. 29415429 10.3390/ijms19020472 PMC5855694

[48] Xu LM Chen L Li F. Over-expression of the long non-coding RNA HOTTIP inhibits glioma cell growth by BRE. J Exp Clin Cancer Res. 2016;35:162. 27733185 10.1186/s13046-016-0431-y PMC5062847

[49] Yuan Q Liu Y Fan Y. LncRNA HOTTIP promotes papillary thyroid carcinoma cell proliferation, invasion and migration by regulating miR-637. Int J Biochem Cell Biol. 2018;98:1–9. 29474928 10.1016/j.biocel.2018.02.013

[50] Zhang H Zhao L Wang YX Xi M Liu SL Luo LL. Long non-coding RNA HOTTIP is correlated with progression and prognosis in tongue squamous cell carcinoma. Tumour Biol. 2015;36:8805–9. 26058875 10.1007/s13277-015-3645-2

[51] Zhang S Wang W Liu G. Long non-coding RNA HOTTIP promotes hypoxia-induced epithelial-mesenchymal transition of malignant glioma by regulating the miR-101/ZEB1 axis. Biomed Pharmacother. 2017;95:711–20. 28886531 10.1016/j.biopha.2017.08.133

[52] Sun Y Hu B Wang Q. Long non-coding RNA HOTTIP promotes BCL-2 expression and induces chemoresistance in small cell lung cancer by sponging miR-216a. Cell Death Dis. 2018;9:85. 29367594 10.1038/s41419-017-0113-5 PMC5833383

[53] Sun Y Zhou Y Bai Y. A long non-coding RNA HOTTIP expression is associated with disease progression and predicts outcome in small cell lung cancer patients. Mol Cancer. 2017;16:162. 29041935 10.1186/s12943-017-0729-1 PMC5646126

[54] Sang Y Zhou F Wang D. Up-regulation of long non-coding HOTTIP functions as an oncogene by regulating HOXA13 in non-small cell lung cancer. Am J Transl Res. 2016;8:2022–32. 27347311 PMC4891416

[55] Zhang GJ Song W Song Y. Overexpression of HOTTIP promotes proliferation and drug resistance of lung adenocarcinoma by regulating AKT signaling pathway. Eur Rev Med Pharmacol Sci. 2017;21:5683–90. 29272003 10.26355/eurrev_201712_14013

